# Echocardiographic Assessment of Atrial Function in Patients With Fontan Palliation: Feasibility, Reproducibility, and Prognostic Implications

**DOI:** 10.1016/j.cjcpc.2025.02.002

**Published:** 2025-03-08

**Authors:** Sara ElZalabany, Amr Moustafa, Ali Ali, Ahmed T. Abdelhalim, Ahmed E. Ali, Luke J. Burchill, Maan Jokhadar, Alexander C. Egbe

**Affiliations:** Department of Cardiovascular Medicine, Mayo Clinic, Rochester, Minnesota, USA

## Abstract

**Background:**

Atrial strain provides a global assessment of left heart diastolic function in patients with biventricular circulation, and it is used for risk stratification. However, the role of atrial strain imaging for risk stratification in patients with Fontan palliation has not been studied, and this is likely related to the complex anatomy of the pulmonary venous atrium in this population. The purpose of this study was to assess the feasibility and reproducibility of echocardiographic indices of pulmonary venous atrial function and their relationship to clinical outcomes.

**Methods:**

This is a retrospective cohort study of adults with Fontan palliations who underwent transthoracic echocardiogram at Mayo Clinic (2003-2023). Atrial reservoir strain was used as a measure of global atrial function and was assessed using speckle tracking imaging. The relationship between atrial reservoir strain and death/transplant was assessed using multivariable Cox regression analysis.

**Results:**

Of 518 patients, the assessment of atrial strain was feasible in 411 (79%), with modest intraobserver and interobserver reproducibility (intraclass correlation: 0.83, 95% confidence interval [CI]: 0.76-0.89 and intraclass correlation: 0.81, 95% CI: 0.74-0.87, respectively). The correlates of atrial dysfunction (worse atrial reservoir strain) were older age, systemic ventricular systolic dysfunction, and history of atrial fibrillation. There was a 13% decrease in the risk of death/transplant for every 5% increase in atrial reservoir strain (adjusted hazard ratio: 0.87, 95% CI: 0.72-0.92, *P* = 0.02) after adjustment for demographic indices, surgical/anatomic indices, and comorbidities/end-organ function.

**Conclusions:**

Echocardiographic assessment of pulmonary venous atrial strain was feasible and reproducible and can be used for risk stratification in adults with Fontan palliation.

## Résumé

**Contexte:**

La déformation auriculaire permet une évaluation globale de la fonction diastolique du cœur gauche chez les patients présentant une circulation biventriculaire et est utilisée pour stratifier le risque. Cependant, le rôle joué par l’imagerie de la déformation auriculaire dans la stratification du risque chez les patients subissant l’intervention de Fontan n’a pas fait l’objet d’études, ce qui est probablement dû à l’anatomie complexe de l’oreillette gauche au sein de cette population. Cette étude visait à évaluer la faisabilité et la reproductibilité des indices échocardiographiques de la fonction de l’oreillette gauche ainsi que leur lien avec les issues cliniques.

**Méthodologie:**

Il s’agit d’une étude de cohorte rétrospective menée auprès d’adultes qui ont subi une intervention de Fontan et qui ont été soumis à un échocardiogramme transthoracique dans une clinique Mayo (2003-2023). La déformation de l’oreillette a été utilisée comme mesure de la fonction auriculaire globale et a été évaluée à l’aide de l’imagerie de suivi des marqueurs acoustiques. Le lien entre la déformation auriculaire et le décès ou la greffe a été évalué par une analyse de régression de Cox multivariée.

**Résultats:**

La déformation auriculaire a pu être évaluée chez 411 des 518 patients (79 %), avec une reproductibilité intraobservateur et interobservateur modeste (corrélation intraclasse : 0,83, intervalle de confiance [IC] à 95 % : 0,76-0,89 et corrélation intraclasse : 0,81, IC à 95 % : 0,74-0,87, respectivement). Les corrélats de la dysfonction auriculaire (pire déformation auriculaire) étaient l’âge avancé, la dysfonction systolique ventriculaire systémique et des antécédents de fibrillation auriculaire. Le risque de décès ou de greffe était réduit de 13 % pour chaque tranche d’augmentation de 5 % de la déformation auriculaire (rapport de risques instantanés ajusté : 0,87, IC à 95 % : 0,72-0,92, *p* = 0,02) après ajustement en fonction des indices démographiques, des indices chirurgicaux/anatomiques et des maladies concomitantes ou de la fonction des organes cibles.

**Conclusions:**

L’évaluation échocardiographique de la déformation auriculaire étant faisable et reproductible, elle peut être utilisée pour stratifier le risque chez les adultes ayant subi une intervention de Fontan.

Systemic ventricular diastolic dysfunction is common in adults with Fontan palliation, and it is associated with cardiovascular morbidity and mortality.^1^^,^^2^ The early phase of diastolic dysfunction is characterized by impaired systemic ventricular relaxation with minimal change in systemic ventricular filling pressures, and this is followed by impaired systemic ventricular compliance leading to an increase in filling pressures.^3^ The assessment of systemic ventricular filling pressures is challenging in Fontan physiology and typically requires right heart catheterization.^1^^,^^2^^,^^4^ This is because of the inconsistent diagnostic and prognostic performance of the conventional echocardiographic indices of ventricular diastolic function for risk stratification in the Fontan population.^1^^,^^2^

There are limited data about the role of speckle tracking echocardiography for the assessment of pulmonary venous atrial function and the potential application of these indices for risk stratification.^5^^,^^6^ The purpose of this study was to assess the feasibility and reproducibility of echocardiographic indices of pulmonary venous atrial function and their relationship to clinical outcomes. The terms “atrial strain” and “pulmonary venous atrial strain” will be used interchangeably throughout the paper.

## Methods

### Study population

This is a retrospective cohort study of adults (aged ≥18 years) with Fontan palliation who received care at Mayo Clinic from January 1, 2003, to December 31, 2023. The patients were identified from the Mayo Adult Congenital Heart Disease (MACHD) registry, and the Mayo institutional review board approved this study. The first clinical encounter with an echocardiogram after January 1, 2003, was considered as the baseline encounter, and clinical indices obtained within 6 months from the time of baseline encounter were used to define the baseline characteristics.

The patients were categorized based on type of Fontan at the time of baseline echocardiogram into 3 groups: (1) atriopulmonary Fontan, (2) lateral tunnel/intra-atrial conduit Fontan, and (3) extracardiac conduit Fontan. Of note, patients who underwent Fontan conversion operation before the baseline echocardiogram were classified based on the type of Fontan connection at the time of baseline echocardiogram and not the initial type of Fontan connection. We excluded patients with moderate or severe regurgitation of the atrioventricular or prior implantation of atrioventricular valve prosthesis.

We used normal controls from the **P**rospective Observational **R**eg**i**stry of Outcomes in Adults with Congenital Heart Disease at **M**ayo Clinic (PRISM) as a reference group for comparison of atrial function indices. The PRISM registry is a database of adults with congenital heart disease who underwent cardiovascular imaging, cardiopulmonary exercise test, invasive hemodynamic assessment, and biomarker assay from January 1, 2019.

### Study objectives

The objectives of this study were to (1) determine the feasibility and reproducibility of the assessment of indices of atrial function and remodeling, (2) determine the correlates of global atrial function, and (3) assess the relationship between the global atrial function and the composite endpoint of all-cause mortality and/or heart transplant. Outcome was ascertained by a review of electronic health records and the Accurint mortality database from the baseline encounter to the occurrence of composite outcome, last clinical encounter, or December 31, 2023.

### Assessment of atrial structure and function

**Figure 1:**
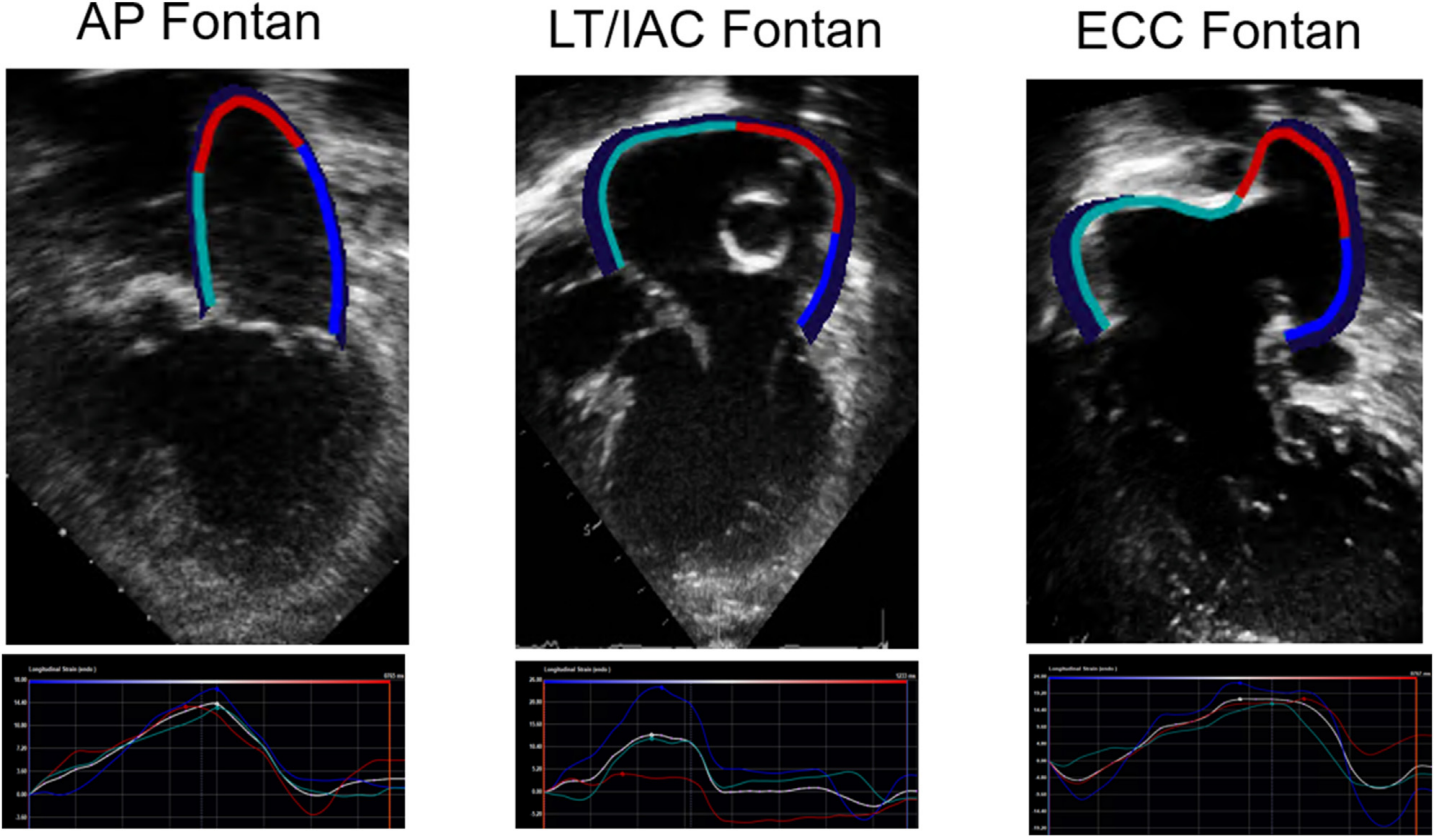
Top: representative echocardiographic images of pulmonary venous atrial strain analyses from a patient with atriopulmonary Fontan (AP), lateral tunnel/intra-atrial conduit (LT/IAC), and extracardiac conduit (ECC) Fontan. Reservoir strain was measured at the end of ventricular systole, booster strain was measured at the end of atrial systole, and conduit strain was calculated as the difference between reservoir strain and booster strain. All strain measurements were made using the QRS as the fiduciary point.

We assessed atrial reservoir strain, conduit strain, and pump strain using speckle tracking echocardiography.^3^^,^^7^^,^^8^ Atrial reservoir strain was dependent on atrial compliance and modulated by ventricular systolic function through descent of the base of the ventricle. Atrial conduit strain was dependent on ventricular relaxation and chamber stiffness. Atrial booster strain was dependent on intrinsic atrial contractility and ventricular end-diastolic compliance. Atrial reservoir strain was considered a measure of global atrial function. Figure 1 shows atrial strain imaging based on type of Fontan connection. Images were obtained using Vivid E9 and E95 (General Electric Co, Fairfield, CT) with M5S and M5Sc-D transducers (1.5-4.6 MHz) at a frame rate of 40-80 Hz, and these images were exported (DICOM) and then analyzed offline using TomTec (TomTec Imaging Systems, Unterschleissheim, Germany). Atrial strain was assessed using a 3-beat cine-loop clips obtained from the apical 4-chamber view, and reservoir strain, conduit strain, and booster strain were assessed using the QRS as the fiduciary point. In patients with atriopulmonary Fontan, we traced the endocardial border of the pulmonary venous atrium, whereas in patients with total cavopulmonary connection (ie, lateral tunnel/intra-atrial conduit Fontan and extracardiac conduit Fontan), we traced the endocardial border of both atria because these patients had a functional common atrium after septectomy. The endocardial border was traced automatically by the software, and the automatic tracings were adjusted manually to ensure optimal tracking throughout the cardiac cycle. Segments were excluded objectively if the software was unable to adequately track the myocardium.

Atrial volume was assessed using 2-dimensional echocardiography from the apical 4-chamber view using the monoplane volumetric method. Maximum and minimum atrial volumes were measured at end-systole and end-diastole, respectively, and atrial ejection fraction was calculated as follows: (maximum atrial volume – minimum atrial volume)/maximum atrial volume × 100. Atrial volumes were index to body surface area.

In addition, systemic ventricular function was assessed using ventricular ejection fraction derived from monoplane volumetric analysis of systemic ventricular end-diastolic and end-systolic volumes measured from apical 4-chamber view. Offline image analysis was performed in all patients by research sonographers using the standard protocol of the MACHD registry imaging core laboratory.^9^

### Statistical analysis

Data were presented as mean ± standard deviation, median (interquartile range), and count (%). Between-group comparisons were performed using the unpaired *t* test, Wilcoxon rank-sum test, analysis of variance tests, or the Fisher exact test, as appropriate. Feasibility analysis for each echocardiographic index of atrial structure and function was estimated as the proportion of patients with measurable indices. Reproducibility analysis was assessed as intraobserver and interobserver agreement in 20 randomly selected samples of patients and expressed as intraclass correlation and 95% confidence interval (95% CI).

The correlates of global atrial function (pulmonary venous atrial reservoir strain) were determined using linear regression analysis. The covariates used in the univariable model were chosen based on clinical relevance, and the correlates with *P* < 0.1 on univariable analysis were used to create a multivariable linear regression model. The final covariate selection was based on stepwise backward selection with *P* < 0.1 required for a covariate to remain in the model.

The association between global atrial function (atrial reservoir strain) and outcomes (death/transplant) was assessed using Cox regression analysis. The Cox regression models were then adjusted for clinical variables (anatomic, surgical, comorbidities/end-organ function, and laboratory indices) using similar criteria for covariate selection as described for the linear regression model. Sensitivity analysis was performed in the subset of patients who were in sinus rhythm at the time of echocardiogram (N = 383) to assess the relationship between atrial reservoir strain and death/transplant using a similar method for Cox regression model adjustments. The cohort was divided into quartiles based on atrial reservoir strain, and the freedom from death/transplant was estimated using the Kaplan-Meier method and compared across the quartiles using the log rank test. All analyses were stratified based on ventricular morphology. All statistical analyses were performed with BlueSky Statistics software (version 7.10; BlueSky Statistics LLC, Chicago, IL) and JMP statistical software (version 17.1.0; JMP Statistical Discovery LLC, Cary, NC). A *P* value of <0.05 was considered to be statistically significant for all analyses.

## Results

### Baseline characteristics

We identified 534 patients with Fontan palliation in the MACHD registry within the study period. Of the 534 patients, 16 (3%) were excluded based on predefined exclusion criteria, and 518 (97%) patients were included in the study. The most common congenital heart lesions were tricuspid atresia (n = 178, 34%), double inlet left ventricle (n = 111, 21%), unbalanced atrioventricular canal defect (n = 63, 12%), hypoplastic left heart syndrome (n = 54, 11%), double-outlet right ventricle (n = 50, 8%), and pulmonary atresia (n = 44, 9%).

Of the 518 patients, 356 (69%) had a morphologic systemic left ventricle. The Fontan connections at initial Fontan operation were atriopulmonary Fontan (n = 329, 64%), lateral tunnel/intra-atrial conduit Fontan (n = 102, 20%), and extracardiac conduit Fontan (n = 87, 17%). Of the 518 patients, 149 (29%) underwent Fontan conversion/revision, and the Fontan connections at the time of baseline echocardiogram were atriopulmonary Fontan (n = 194, 29%), lateral tunnel/intra-atrial conduit Fontan (n = 169, 33%), and extracardiac conduit Fontan (n = 155, 30%) (Table 1).

**Table 1:** Baseline characteristics

Characteristic	All (N = 518)
Age (y)	27 ± 9
Male sex	290 (56)
Body surface area (m^2^)	1.78 ± 0.24
Body mass index (kg/m^2^)	24.5 ± 3.6
Systemic oxygen saturation (%)	92 (89-94)
CIED	126 (24)
Surgical history	
Type of initial Fontan connection	
Atriopulmonary Fontan	329 (64)
Lateral tunnel/intra-atrial conduit Fontan	102 (20)
Extracardiac conduit Fontan	87 (17)
Age at Fontan operation (y)	5 (3-9)
Subsequent Fontan conversion	149 (29)
Maze operation	86 (17)
Fontan connection at baseline echo	
Atriopulmonary Fontan	194 (38)
Lateral tunnel/intra-atrial conduit Fontan	169 (33)
Extracardiac conduit Fontan	155 (30)
Systemic left ventricle	356 (69)
Comorbidities	
Atrial arrhythmias	243 (47)
Atrial flutter/tachycardia	167 (32)
Atrial fibrillation	114 (22)
CKD III-V	16 (4)
Cirrhosis	116 (22)
Protein losing enteropathy	54 (10)
Hypertension	41 (8)
Laboratory data	
NTproBNP (pg/mL)	208 (72-520)
Estimated GFR (mL/min/1.73 m^2^)	103 (82-124)
Hemoglobin (g/dL)	14.9 ± 2.3
MELD-XI	10.4 (9.5-13.1)
Medications	
Diuretics	214 (41)
β–Blockers	182 (35)
ACEI/ARB	308 (60)
MRA	124 (24)
Antiplatelets	269 (52)
Vitamin K antagonist	211 (41)
DOAC	61 (12)
Echocardiographic indices	
Systemic ventricular EDV index (mL/m^2^)	93 (71-124)
Systemic ventricular ESV index (mL/m^2^)	44 (32-84)
Systemic ventricular ejection fraction (%)	54 (47-58)
Doppler-derived ventricular SV index (mL/m^2^)	44 ± 15
Doppler-derived cardiac index, (L/min/m^2^)	3.21 ± 1.08
Systemic AV valve E velocity (m/s)	0.68 ± 0.22
Systemic AV valve A velocity (m/s)	0.48 ± 0.21
Systemic AV valve DT (ms)	166 ± 49
Lateral e′ velocity (cm/s)	10.3 ± 3.69
Lateral (E/e′)	7.58 ± 4.63

Data are presented as mean ± standard deviation, median (interquartile range), and count (%).

ACEI/ARB, angiotensin-converting enzyme inhibitor/angiotensin-II receptor blocker; AV, atrioventricular; CIED, cardiac implantable electronic device; CKD, chronic kidney disease; DOAC, direct acting oral anticoagulant; DT, deceleration time; EDV, end-diastolic volume; ESV, end-systolic volume; GFR, glomerular filtration rate; MELD-XI, model for end-stage liver disease excluding international normalized ratio; MRA, mineralocorticoid receptor antagonist; NTproBNP, N-terminal prohormone of brain natriuretic peptide; SV, stroke volume.

### Atrial structure and function

#### Feasibility and reproducibility

Table 2 shows feasibility and reproducibility of echocardiographic indices of atrial structure and function. Overall, the assessment of atrial reservoir strain, atrial conduit strain, and atrial pump strain was feasible in 411 (79%), 383 (74%), and 383 (74%) patients, respectively. Of the 411 patients, 383 were in sinus rhythm at the time of baseline echocardiogram. There was good intraobserver and interobserver agreement for atrial strain indices (Table 2).

**Table 2:** Feasibility and reproducibility of atrial function indices

	n	Feasibility (%)	Intraobserver ICC (95% CI)	Interobserver ICC (95% CI)
Atrial strain indices				
Atrial reservoir strain (%)	411	79	0.83 (0.76-0.89)	0.81 (0.74-0.87)
Atrial conduit strain (%)	383	74	0.81 (0.77-0.85)	0.80 (0.76-0.84)
Atrial pump strain (%)	383	74	0.82 (0.78-0.86)	0.80 (0.75-0.85)
Atrial volumetric indices				
Atrial maximum volume index (mL/m^2^)	417	81	0.80 (0.74-0.86)	0.78 (0.72-0.84)
Atrial minimum volume index (mL/m^2^)	415	80	0.79 (0.74-0.84)	0.78 (0.73-0.83)

Feasibility was calculated as the number of patients with available/measurable data (n) divided by the total number of patents (N = 518) × 100.

CI, confidence interval; ICC, intraclass correlation coefficient.

Table 3 shows between-group comparison of echocardiographic indices of atrial size and function. Compared with the control group, the Fontan group had lower atrial reservoir strain (17% [12%-24%] vs 38% [31%-42%], *P* < 0.001), atrial conduit strain (12% [8%-16%] vs 28% [23%-35%], *P* < 0.001), and atrial pump strain (5% [2%-9%] vs 9% [4%-11%], *P* = 0.006). Similarly, the Fontan group had lower atrial ejection fraction (42% [30%-51%] vs 61% [52%-70%], *P* < 0.001) but comparable maximum atrial volume index (38 [20-44] vs 25 [23-32] mL/m^2^, *P* = 0.2) (Table 3).

**Table 3:** Atrial structure and function

	Controls (N = 41)	All Fontan (N = 411)	AP Fontan (N = 149, 36%)	LT/IAC Fontan (N = 136, 33%)	ECC Fontan (N = 126, 31%)	*P*
Demographic indices						
Age (y)	42 ± 14∗	27 ± 9∗	29 ± 9	28 ± 8	25 ± 8	<0.001
Atrial strain indices (%)						
Atrial reservoir strain	38 (31-42)∗	17 (12-24)∗	17 (11-24)	18 (13-18)	18 (13-18)	0.3
Atrial conduit strain	28 (23-35)∗	12 (8-16)∗	11 (7-15)	12 (8-17)	13 (9-18)	0.06
Atrial pump strain	9 (4-11)∗	5 (2-9)∗	5 (2-9)	5 (3-10)	4 (2-8)	0.7
Atrial volumetric indices						
Maximum atrial volume index (mL/m^2^)	25 (23-32)	28 (20-44)	30 (21-41)	28 (20-44)	27 (19-49)	0.8
Minimum atrial volume index (mL/m^2^)	11 (7-16)∗	16 (11-28)∗	17 (12-27)	16 (11-28)	16 (9-32)	0.8
Atrial ejection fraction (%)	61 (52-70)∗	42 (30-51)∗	42 (27-50)	42 (32-51)	44 (31-53)	0.4

Data are presented as mean ± standard deviation and median (interquartile range).

Comparisons between Fontan and controls were based on the unpaired *t* test or the Wilcoxon rank-sum test as appropriate.

*P* value was based on comparison across the 3 subgroups showing the type of Fontan connection at the time of baseline echocardiogram.

AP, atriopulmonary; ECC, extracardiac conduit; LT/IAC, lateral tunnel/intra-atrial conduit.

∗ Signify statistically significant difference between Fontan and controls.

Within the Fontan group, there was no significant difference in atrial strain, atrial volumes, or atrial ejection fraction based on the type of Fontan connection at the time of echocardiogram (Table 3).

#### Correlates of atrial reservoir strain

On multivariable analysis, the correlates of atrial reservoir strain were age (β ± standard error [SE]: –0.47 ± 0.21 per 5 years, *P* = 0.04), systemic ventricular ejection fraction (β ± SE: 0.67 ± 0.18 per 5%, *P* < 0.001), history of atrial fibrillation (β ± SE: –2.18 ± 1.01, *P* = 0.03), and atrial ejection fraction (β ± SE: 0.61 ± 0.22 per 5%, *P* = 0.003) (Table 4).

**Table 4:** Linear regression analysis assessing for correlates of atrial reservoir strain

	Univariable model		Multivariable model	
	β ± SE	*P*	β ± SE	*P*
Demographic indices				
Age, per 5 years	–1.00 ± 0.22	<0.001	–0.47 ± 0.21	0.04
Male sex	0.21 ± 0.39	0.6		
Body mass index, per 5 kg/m^2^	–0.06 ± 0.14	0.9		
Surgical data				
Fontan connection at initial Fontan				
AP Fontan	Reference	–	Reference	–
LT/IAC Fontan	2.78 ± 1.00	0.005	1.71 ± 1.00	0.09
ECC Fontan	3.33 ± 1.02	0.001	1.11 ± 1.06	0.30
Age at Fontan operation, per year	–0.05 ± 0.06	<0.001		
Fontan conversion operation	–2.51 ± 0.85	0.003	–1.76 ± 0.99	0.08
Fontan connection at baseline echocardiogram				
AP Fontan	Reference	–		
IAC/LTF	0.82 ± 0.94	0.4		
ECF	0.94 ± –0.95	0.3		
History of atrial maze	–0.64 ± 0.28	0.008		
Paced rhythm	–0.83 ± 0.46	0.07		
Atrial remodeling/arrhythmia				
Max atrial volume index, per 5 mL/m^2^	–0.26 ± 0.08	<0.001		
Atrial EF, per 5%	0.87 ± 0.13	<0.001	0.61 ± 0.22	0.003
Atrial arrhythmias				
No prior atrial arrhythmias	Reference	–	Reference	–
History of atrial flutter alone	–1.43 ± 0.85	0.09	–0.49 ± 0.83	0.5
History of atrial fibrillation	–4.27 ± 0.96	<0.001	–2.18 ± 1.01	0.03
Systemic ventricle				
Systemic left ventricle	–1.26 ± 0.85	0.1		
Systemic ventricular EF, per 5%	0.76 ± 0.18	<0.001	0.67 ± 0.18	<0.001

Multivariable model was created by stepwise backward selection of covariates as described in the Statistical analysis subsection.

AP, atriopulmonary; ECC, extracardiac conduit; ECF, extracardiac conduit Fontan; EF, ejection fraction; IAC, intra-atrial conduit; LT, lateral tunnel; SE, standard error.

### Prognostic implications of atrial reservoir strain

**Figure 2:**
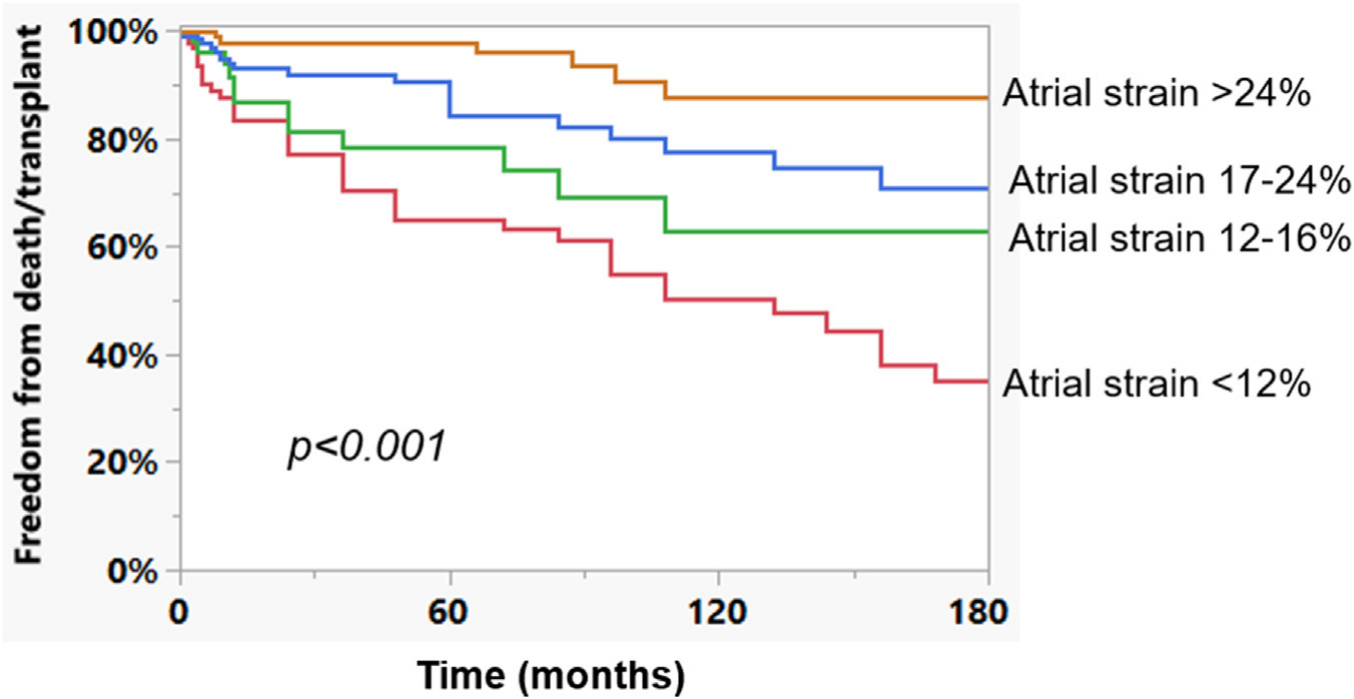
Kaplan-Meier curves comparing freedom from death/transplant across the quartiles of atrial reservoir strain.

Of the 411 patients, 91 (22%) died and 19 (5%) underwent heart transplant. The composite outcome of death/transplant occurred in 104 (23%), and the 10- and 15-year freedom from death/transplant was 72% and 64%, respectively. The 15-year freedom from death/transplant across the quartiles of atrial reservoir strain is shown in Figure 2. The 15-year freedom from death/transplant ranged from 88% in the top quartile to 36% in the bottom quartile of atrial reservoir strain (Fig. 2). There was a 13% decrease in the risk of death/heart transplant for every 5% increase in atrial reservoir strain (adjusted hazard ratio [HR]: 0.87, 95% CI: 0.72-0.92, *P* = 0.02), after adjustment for demographic indices, surgical/anatomic indices, and comorbidities/end-organ function (Table 5). The other clinical and echocardiographic indices associated with death/transplant were systemic ventricular ejection fraction (adjusted HR: 0.86, 95% CI: 0.77-0.97 per 5%, *P* = 0.01), cardiac implantable electronic device (adjusted HR: 2.39, 95% CI: 1.35-4.69, *P* = 0.003), cirrhosis (adjusted HR: 2.78, 95% CI: 1.47-5.28, *P* = 0.002), protein-losing enteropathy (adjusted HR: 2.13, 95% CI: 1.04-3.37, *P* = 0.04), and hepatorenal function as measured by a model for end-stage liver disease excluding international normalized ratio (adjusted HR: 1.11, 95% CI: 1.05-1.19, *P* < 0.011) (Table 5).

**Table 5:** Cox regression models for correlates of death/transplant

	Univariable		Multivariable	
	HR (95% CI)	*P*	HR (95% CI)	*P*
Atrial indices				
Atrial reservoir strain, per 5%	0.73 (0.62-0.82)	<0.001	0.87 (0.72-0.92)	0.02
Atrial EF, per 5%	0.88 (0.82-0.94)	0.002		
Max atrial volume index, per 5 mL/m^2^	1.10 (1.06-1.15)	<0.001		
Ventricular indices				
Ventricular EF, per 5%	0.82 (0.77-0.90)	<0.001	0.86 (0.77-0.97)	0.01
Lateral e′ (cm/s)	1.6 (0.68-2.95)	0.7		
Lateral E/e′	1.01 (0.90-1.06)	0.8		
Demographic/anatomic indices				
Age, per 5 years	1.27 (1.17-1.38)	<0.001	1.03 (0.87-1.21)	0.2
Male sex	1.10 (0.78-1.56)	0.6	1.08 (0.72-1.66)	0.7
AP Fontan at initial Fontan operation	2.03 (1.27-3.26)	0.003		
Age at Fontan operation, per year	1.03 (0.94-1.12)	0.4		
AP Fontan at baseline echocardiogram	1.34 (0.96-1.90)	0.09		
CIED	2.17 (1.51-3.15)	<0.001	2.39 (1.35-4.69)	0.003
Comorbidities/end-organ dysfunction				
Atrial fibrillation	2.60 (1.83-3.69)	<0.001		
Atrial flutter	1.23 (0.86-1.76)	0.3		
Cirrhosis	2.31 (1.53-3.48)	<0.001	2.78 (1.47-5.28)	0.002
Protein losing enteropathy	2.57 (1.68-3.94)	<0.001	2.13 (1.04-4.37)	0.04
Type 2 diabetes	2.05 (0.91-4.68)	0.08		
CKD III-V	4.56 (2.37-8.79)	<0.001		
Laboratory indices				
GFR, per 10 mL/min/1.73 m^2^	0.86 (0.79-0.91)	0.003		
Log NTproBNP (pg/mL)	1.53 (1.27-1.85)	<0.001	1.21 (0.99-1.49)	0.07
MELD-XI	1.12 (1.09-1.16)	<0.001	1.11 (1.05-1.19)	<0.001

Multivariable model was created by stepwise backward selection of covariates as described in the Statistical analysis subsection.

AP, atriopulmonary; CI, confidence interval; CIED, cardiac implantable electronic device; CKD, chronic kidney disease; EF, ejection fraction; GFR, glomerular filtration rate; HR, hazard ratio; MELD-XI, model for end-stage liver disease excluding international normalized ratio; NT-proBNP, N-terminal prohormone of brain natriuretic peptide.

#### Sensitivity analysis

Sensitivity analysis was performed to assess the prognostic implication of atrial reservoir strain in the 383 patients who were in sinus rhythm at the time of echocardiogram. In this analysis, atrial reservoir strain was also associated with death/heart transplant (adjusted HR: 0.89, 95% CI: 0.73-0.94, *P* = 0.03), after adjustment for demographic indices, surgical/anatomic indices, and comorbidities/end-organ function.

## Discussion

In this study, we assessed the feasibility, reproducibility, and prognostic value of echocardiographic indices of atrial reservoir strain in patients with Fontan palliation. The main findings are as follows: (1) the assessment of echocardiographic indices of atrial reservoir strain was feasible in 74% of patients, with modest intraobserver and interobserver reproducibility; (2) the correlates of atrial dysfunction (worse atrial reservoir strain) were older age, systemic ventricular systolic dysfunction, and history of atrial fibrillation; and (3) atrial dysfunction was associated with a higher risk of death/transplant, independent of other clinical and echocardiographic risk factors.

In patients with biventricular circulation, the left atrium modulates left ventricular preload and global diastolic function.^3^^,^^8^^,^^10^ Speckle tracking echocardiography provides a robust assessment of left atrial function.^3^^,^^8^^,^^10^ Left atrial reservoir strain, derived from speckle tracking echocardiography, has been shown to correlate with the risk of atrial fibrillation, heart failure, and cardiovascular mortality.^3^^,^^8^^,^^10^ As a result, left atrial reservoir strain has been used for risk stratification in patients with acquired heart disease.^3^^,^^8^^,^^10^ Similar data are sparse in the adult congenital heart disease population, but emerging data suggest that atrial reservoir strain can be used for risk stratification in patients in this population.^11^, ^12^, ^13^ Veldtman et al.^5^ compared pulmonary venous atrial strain in 39 pediatric patients with Fontan palliation with age-matched controls and observed a lower atrial strain in the Fontan group. In a different study, Rato et al.^6^ demonstrated that patients with Fontan palliation had lower pulmonary venous atrial reservoir strain, and that pulmonary venous atrial strain was associated with peak oxygen consumption. The current study builds on this foundation and provides new insight into the role of atrial strain for risk stratification in the Fontan population. First, the current study identified correlates of atrial dysfunction that include older age, atrial fibrillation, and systemic ventricular systolic dysfunction. Furthermore, the current study demonstrated an association between atrial dysfunction and mortality in the Fontan population. This is consistent with emerging data from the congenital heart disease population highlighting the prognostic implication of atrial dysfunction and the potential role of atrial strain for risk stratification in this population.^11^, ^12^, ^13^ In addition, other studies have assessed atrial function in pediatric Fontan patients.^14^^,^^15^ In a cross-sectional study, Khoo et al.^14^ assessed atrial function using speckle tracking echocardiography in 81 pediatric patients, of whom only 14 patients had Fontan completion. They observed that the patients had larger atrial size and lower atrial reservoir strain than healthy controls.^14^ However, the mean age of the 14 Fontan patients was 4.4 years, and this makes it challenging to make any form of meaningful comparison with the current study (mean age 27 years) considering age-related differences in cardiac remodeling as well as comorbidities in the current cohort. In another study, van der Ven et al.^15^ assessed the relationship between atrial function as measured by cardiac magnetic resonance imaging and exercise capacity in pediatric patients with total cavopulmonary connection. Atrial reservoir strain was not associated with aerobic capacity as measured by peak oxygen consumption and with a composite adverse outcome in that study.^15^ This contrasts with the current study showing an association between atrial reservoir strain and death/transplant. The observed differences may be related to differences in demographic characteristics (younger Fontan cohort with total cavopulmonary connection vs older Fontan cohort with multiple comorbidities, of which more than half of the cohort had prior atriopulmonary connection) and differences in event rate (2% mortality vs 22% mortality).^15^ While prior studies examined the role of atrial function in the Fontan population, the current study presents data about the prognostic role of atrial function in a high-risk Fontan cohort.^14^^,^^15^ As the Fontan patients become older and develop more comorbidities, prognostic data based on a higher risk population such as the current study will be required for risk stratification.

Despite improvements in medical and surgical management of adults with Fontan palliation, the long-term survival of adults with Fontan palliation remains significantly lower than that of the general population and other patients with congenital heart disease.^4^^,^^16^^,^^17^ To improve outcomes in the Fontan population, there is a need to develop robust clinical tools to identify patients at risk for adverse outcomes and to treat the modifiable risk factors in such patients or refer the patient for heart transplantation. The result of the current study suggests that reduced atrial reservoir strain may be used to identify patients at risk for mortality, which in turn may provide opportunity to treat modifiable risk factors or refer such patients for heart transplantation.

### Limitations

This is a retrospective single-center study at a tertiary center, and it is prone to selection and ascertainment bias. The atrial strain data were based on offline analysis by research sonographers using a standardized imaging core lab protocol. As a result, the feasibility, reproducibility, and correlation estimates observed in this study may overestimate the expected results when applied in routine clinical practice.

## Conclusions

The assessment of atrial strain in adults with Fontan palliation was feasible and reproducible, and atrial strain was prognostic in this population. Reduced atrial reservoir strain may potentially be used to identify patients at risk for mortality, which in turn may provide opportunity for intensification of therapy or referral for heart transplant evaluation. Further studies are required to determine whether these postulates regarding clinical application of atrial strain are supported by empirical data.
